# Porcine blood and surrogate markers do not prove benefit of aDabi-Fab

**DOI:** 10.1186/cc13927

**Published:** 2014-06-17

**Authors:** Nicholas J Connors, Justin Gill, Allison Nakanishi, Robert S Hoffman

**Affiliations:** 1 Division of Medical Toxicology, Department of Emergency Medicine, New York University School of Medicine, 450 First Avenue, Room 123, New York NY 10016, USA; 2 University of British Columbia Faculty of Medicine, 317–2194 Health Sciences Mall, Vancouver BC V6T 1Z3, Canada

## 

While we read with great interest the recent report concerning the reversal of dabigatran-induced coagulopathy [1], we are concerned that methodological issues limit the validity of the authors’ conclusions. Firstly, it is unclear why a porcine model was utilized. Although there is clinical relevance for testing reversal agents in a trauma model, all agents were administered *ex vivo*. Furthermore, the established correlation between the activated partial thromboplastin time (aPTT) and dabigatran concentrations in humans [2] is absent in the pig model. Potentially, porcine coagulation parameters respond to dabigatran and its reversal differently than humans. Most importantly, only surrogate markers of coagulation were reported. Having created a hemorrhagic shock model, the authors should have tested either survival or blood loss following *in vivo* reversal along similar lines of investigation as their prior model [3].

Since further study is needed to determine the usefulness of aDabi-Fab, we would recommend that *ex vivo* studies use human blood while animal studies focus on endpoints that have clear clinical relevance.

## Authors’ response

Oliver Grottke, Joanne van Ryn, Henri MH Spronk and Rolf Rossaint

We thank the authors for their interest in our article [1]. We chose a porcine model to facilitate investigation of dabigatran reversal using several different agents at different concentrations, in blood samples taken both before and after trauma. This method was chosen mainly for ethical reasons, to provide a basis for subsequent *in vivo* studies. *Ex vivo* studies in humans were considered, but in that setting blood would probably be spiked with dabigatran and the effects of coagulation status changes following trauma (for example, hypofibrinogenemia) could not be evaluated. Clinically relevant endpoints such as blood loss are clearly more important than laboratory data and results from a porcine model may not be reflected in human patients. These limitations are acknowledged within our article.

Our article [1] presents our first investigations of dabigatran reversal, including the preliminary dose-finding work required to proceed with *in vivo* studies. Using a similar porcine model of trauma, we now have initial *in vivo* results [4]. A prothrombin complex concentrate dose of 50 IU/kg was effective in reducing total blood loss and increasing survival. This indicates that our *ex vivo* data are applicable *in vivo*.

We disagree that correlation between aPTT and dabigatran concentration is absent in the porcine model. Sensitivity to dabigatran may be different in porcine versus human plasma, and our data suggest that the aPTT-dabigatran concentration curve is less steep in pigs than in humans when using actin FS (Dade Behring, Germany). However, as shown by the results of our study [1], aPTT is prolonged with increasing levels of dabigatran in pigs.

## Abbreviations

aPTT: activated partial thromboplastin time.

## Competing interests

OG has received research funding from Novo Nordisk, Biotest, CSL Behring, Nycomed. He has also received honoraria for consultancy and/or travel support from Bayer Healthcare, Boehringer Ingelheim and CSL Behring. RR has received honoraria for lectures and consultancy from CSL Behring and Novo Nordisk. JvR is an employee of Boehringer Ingelheim Pharma GmbH & Co., Germany. HMHS has received research funding from Boehringer Ingelheim and honoraria for consultancy from Bayer.
